# Plasma and MRI biomarkers capture neuronal damage in former professional boxers

**DOI:** 10.1038/s41598-025-93066-6

**Published:** 2025-06-06

**Authors:** Suzie Kamps, Suzan van Amerongen, Elena R. Blujdea, Marloes I. Hoppen, Bram Bongers, Kasper H. Schelvis, Dewi K. Caton, Marsh Königs, Charlotte E. Teunissen, Philip Scheltens, Rik Ossenkoppele, Everard G. B. Vijverberg

**Affiliations:** 1 Alzheimer Center Amsterdam, Neurology, Vrije Universiteit Amsterdam, Amsterdam UMC location VUmc, Amsterdam, The Netherlands; 2 Amsterdam Neuroscience, Neurodegeneration, Amsterdam, The Netherlands; 3 Neurochemistry Laboratory, Department of Laboratory Medicine, Amsterdam Neuroscience, Amsterdam UMC, Vrije Universiteit Amsterdam, Amsterdam, The Netherlands; 4 Department of Pediatrics, Emma Children’S Hospital, Amsterdam UMC Location University of Amsterdam, Amsterdam, The Netherlands; 5 Emma Neuroscience Group, Emma Children’S Hospital, Amsterdam UMC Location University of Amsterdam, Amsterdam, The Netherlands; 6 Amsterdam Reproduction and Development Research Institute, Amsterdam, The Netherlands; 7 European Clinical Research Alliance on Infectious Diseases, Utrecht, The Netherlands; 8 Emma Children’s Hospital, Amsterdam UMC Location University of Amsterdam, Follow-Me Program, Amsterdam, The Netherlands; 9 Dementia Fund, EQT Life Sciences, Amsterdam, The Netherlands; 10 Department of Clinical Sciences, Lund University, Lund, Sweden

**Keywords:** Boxing, Repetitive head impacts, Plasma biomarkers, Neuroimaging, Neuronal damage, Chronic traumatic encephalopathy, Neurodegeneration, Contact sports, Biomarkers, Dementia, Neurodegeneration, Brain injuries, Dementia, Neurodegeneration, Neurodegenerative diseases

## Abstract

Repetitive Head Impacts (RHI) in boxing may increase the risk for neuronal damage. We evaluated blood-based biomarkers for neurodegeneration and Alzheimer’s Disease (AD) pathology, clinical features, and MRI biomarkers in former professional boxers compared to patients with AD or patients with subjective cognitive decline (SCD). Nine former boxers were compared to age-matched patients (without single TBI or RHI) with biomarker confirmed AD (*N* = 15) or SCD (*N* = 14). Plasma samples were analyzed for amyloid beta (Aβ)42, Aβ42/40 ratio, phosphorylated tau (p-tau)181, p-tau217, glial fibrillary acidic protein (GFAP) and neurofilament light (NfL) concentrations. Brain MRI images were processed through Freesurfer to compute disease-related regions-of-interest (ROIs) for AD, frontotemporal dementia (FTD), or chronic traumatic encephalopathy (CTE). Cognitive performance was assessed in memory and executive functioning (EF) domains. Five boxers had follow-up measurements after ~ 7 years. Former boxers had higher plasma GFAP, p-tau181 and p-tau217 than patients with SCD, lower volumes in all pre-specified disease-related brain regions. No differences on neuropsychological test scores between boxers and those with SCD were observed. Longitudinal analysis in the boxer group demonstrated elevation of plasma NfL and p-tau181 over time. The current study suggests the presence of biological processes indicative for neurodegeneration in former boxers, without evident clinical decline. Plasma GFAP, p-tau181 and p-tau217 may be promising biomarkers for indicating risk for neuronal damage following RHI.

**Supplementary Information:**

The online version contains supplementary material available at 10.1038/s41598-025-93066-6.

## Background

Boxing coincides with repetitive head impacts (RHI) and is a risk factor for neurodegenerative disease, notably chronic traumatic encephalopathy (CTE)^1–5^. Neuropathological criteria to diagnose CTE post-mortem are well established^3^, including perivascular depositions of hyperphosphorylated tau (p-tau) at the depths of cortical sulci^4^. CTE cannot be diagnosed during life due to a lack of specific validated biomarkers^2^. Since CTE-pathology often co-occurs with AD-pathology and there is prominent clinical overlap^2^, finding in vivo biomarkers for CTE remains challenging.

A growing body of literature focuses on plasma and imaging biomarkers that potentially reflect long-term damage related to exposure to RHI^6–12^. Biomarkers of interest in plasma include phosphorylated tau at threonine 181 (p-tau181) or 217 (p-tau217)^7,11,12^, neurofilament light protein (NfL), glial fibrillary acidic protein (GFAP)^9,13,14^and the 1–42 peptide of amyloid beta (Aβ42)^6,8^. Aβ and p-tau indicate the accumulation of amyloid and tau in the brain, which are hallmark features of Alzheimer’s Disease (AD) pathology, and the tau isoforms of p-tau181 and p-tau217 have been shown to be elevated in (retired) contact sports athletes^11,12^. NfL and GFAP are markers of axonal damage and astrocytic activation, respectively, and are linked to traumatic brain injury and neurodegeneration^2,10,15^. As these biomarkers are involved in the similar pathological mechanisms of neurodegeneration and TBI, they are described as candidate fluid biomarkers for RHI-related neurodegeneration such as CTE^2^. Development of ultrasensitive assays allows the measurement of these biomarkers in plasma and provides a minimally invasive approach in assessing neurodegenerative processes in individuals exposed to RHI, which is important for a better characterization of CTE pathophysiology. Imaging biomarkers associated with RHI include a cavum septum pellucidum (CSP) and atrophy in subcortical regions^16–18^.

Few studies have evaluated multiple biomarkers in individuals exposed to RHI or compared these biomarkers against other neurodegenerative diseases^12,19,20^. Moreover, previous studies often contain heterogeneous cohorts (e.g. mixed contact sports), and have yielded inconsistent results in terms of biomarker outcomes^6,8,19–21^. For the current study we set out to capture neurodegenerative changes using plasma and imaging biomarkers in former professional boxers that had substantial RHI-exposure. We compared these with results for memory clinic patients with AD or cognitively normal individuals without AD, i.e. Subjective Cognitive Decline (SCD), to evaluate neurodegenerative risk. We expected boxers to show increased levels of plasma biomarkers and evidence for atrophy on MRI in comparison to those with SCD, but not compared to patients with AD.

## Results

### Sample characteristics

Descriptive statistics are provided in Table 1. Across the groups (Boxers, *N* = 9; SCD, *N* = 14; AD, *N* = 15) participants had a mean age of 57.91 years (*SD *= 6.65) and a mean education level of 4.97 (*SD* = 1.26, range 2–7). Groups did not differ on elevated risk for neurodegeneration based on familial cardiovascular, neurodegenerative or psychiatric disease. These potential risk factors are provided in Supplementary Table S1. ANOVAs between groups for age showed boxers (*N* = 9) and patients with SCD (*N* = 14) did not differ significantly in age (*p* = .933), patients with AD (*N* = 15) were significantly older than both boxers (*p* = .021) and patients with SCD (*p* = .013). Boxers had lower education levels than patients with SCD (*p* = .011), but not compared to patients with AD (*p* = .766). Ethnicity is not described as this information was missing for nearly all individuals of the comparison sample, and although for the boxers it was known, this information could lead to identification of these subjects. MMSE scores were similar for boxers and patients with SCD (*p* = .660) and lowest in patients with AD compared to boxers and patients with SCD (both *p* < .001). The average exposure in years was 30.6 years (*SD* = 16.05), with a mean number of rounds fought of 173.63 (*SD* = 100.08), the average number of competitions was 138.63 (*SD* = 80.14) and the mean number of knockouts (KOs) during the professional careers was 15.00 (*SD* = 11.93). None of the boxers had abnormal CSF Aβ values. Five boxers did not have evident cognitive impairment and were therefore classified as SCD, four boxers had amnestic MCI based on neuropsychological assessment. For the first visit, five boxers were classified as having no TES, one as suggestive of TES, one with possible TES and two with probable TES. After follow-up, one boxer was classified with no TES, one boxer as suggestive of TES, and three boxers were classified as having possible TES. Three out of five boxers progressed in their TES classification. Linear regression analyses in the overall study sample showed significant effects of age on plasma Aβ42/40 ratio (*p* < .01), NfL (*p* < .01), and GFAP (*p* < .01) levels, but not on the other plasma biomarkers. Linear regression analyses within boxers showed no significant effects of age on plasma biomarker levels (*p* > .05). Patient groups (AD, SCD, boxers) did not differ significantly in BMI (*p* = .746).

**Table 1:** Descriptive statistics of the study sample.

	Boxers	SCD	AD	*p*-value
N	9	14	15	
Age (mean (SD))	55.67 (7.70)	54.79 (5.55)	62.07 (4.79)	0.004
Level of Education* (mean (SD))	4.22 (1.20)	5.57 (1.22)	4.87 (1.13)	0.035
BMI (mean (SD))	28.10 (4.58)	26.63 (4.26)	27.24 (4.58)	0.746
MMSE (mean (SD))	27.00 (1.00)	28.07 (1.77)	20.40 (4.14)	< 0.001
GDS (mean (SD))	1.50 (2.33)	3.64 (2.24)	2.23 (1.59)	0.054
APOE-E4 carrier (%)	2 (25%)	3 (21.4%)	13 (86.6%)	0.005
Aβ40 pg/mL (mean (SD))	100.95 (13.46)	94.21 (15.41)	103.05 (22.39)	0.413
Aβ42 pg/mL (mean (SD))	6.73 (0.69)	6.00 (1.00)	5.64 (1.65)	0.130
Aβ42/40 ratio (mean (SD))	0.07 (0.01)	0.06 (0.01)	0.05 (0.01)	< 0.001
NfL pg/mL (mean (SD))	12.41 (5.70)	8.42 (3.07)	18.99 (6.42)	< 0.001
GFAP pg/mL(mean (SD))	82.51 (39.73)	39.90 (17.91)	129.25 (62.88)	< 0.001
p-tau181 pg/mL (median [IQR])**	2.21 [1.83, 2.52]	1.50 [1.17, 1.70]	3.19 [2.56, 4.08]	< 0.001
p-tau217 pg/mL (median [IQR])**	0.47 [0.35, 0.58]	0.23 [0.19, 0.37]	0.98 [0.74, 1.28]	< 0.001
NPI frequency total (mean (SD))***	4.33 (5.86)	7.86 (6.47)	5.89 (9.80)	0.712
NPI severity total (mean (SD))***	2.33 (2.52)	6.07 (5.05)	4.22 (6.06)	0.473
Exposure in years (mean (SD))	26.89 (16.05)			
KOs (mean (SD))	15.00 (11.93)			

MMSE = Mini Mental State Examination; Aβ = amyloid beta; GFAP = Glial Fibrillary Acidic Protein; NfL = neurofilament light ; p-tau = phosphorylated tau; NPI = neuropsychiatric inventory; SCD = subjective cognitive decline; AD = Alzheimer’s disease; APOE = Apolipoproteine E; CDR = Clinical Dementia Rating Scale; pg = picogram; GDS = Geriatric Depression Scale; BMI = Body Mass Index; IQR = Inter Quartile Range; SD = Standard Deviation; KO = Knockout

*Level of education is indicated by the Verhage scale^51^

**Medians and IQRs are given where values are not normally distributed.

***NPI scores were only available for three of the boxers

## Plasma biomarkers

**Fig. 1:**
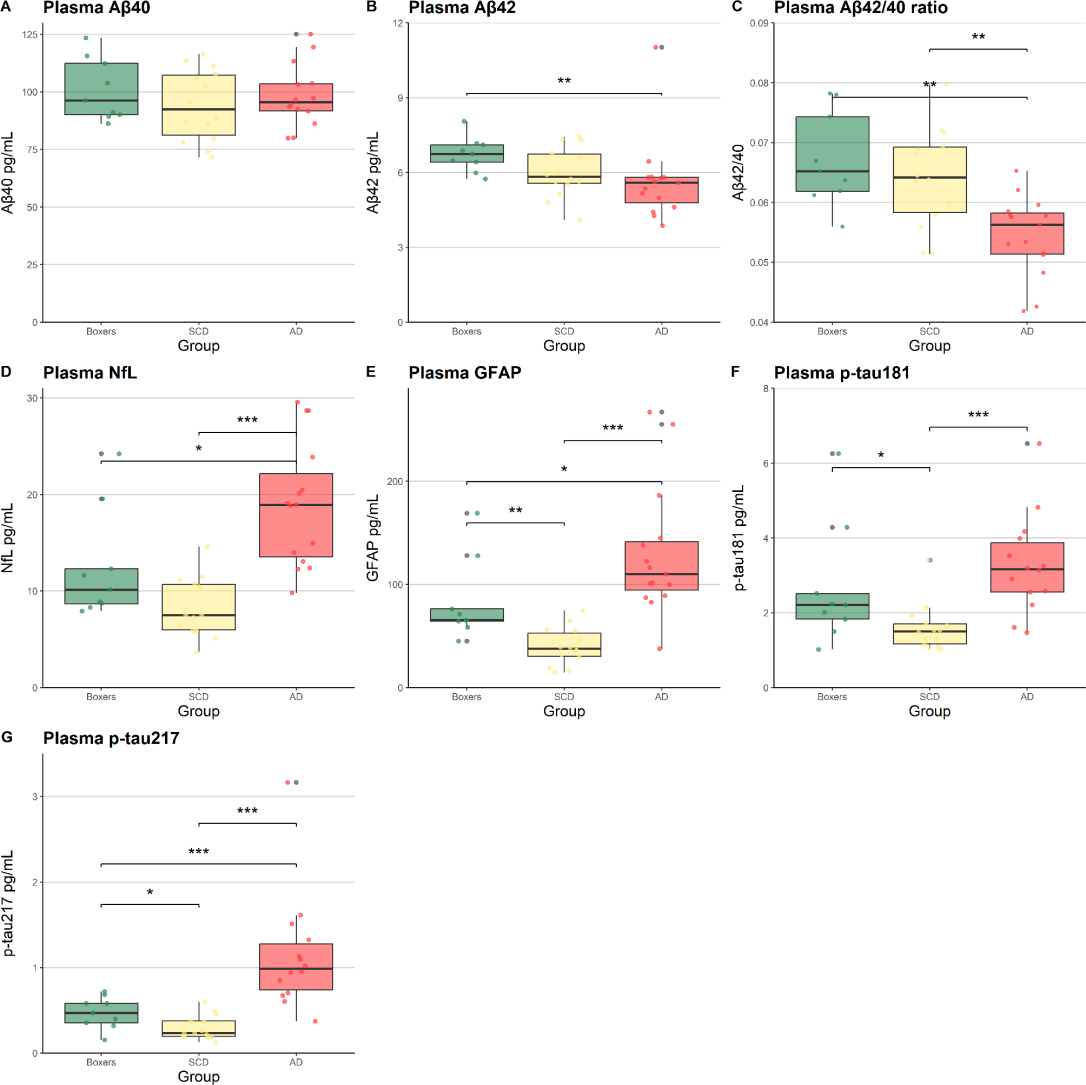
Plasma biomarker concentrations for each group (AD, SCD, boxers). Boxplot depicting the medians and interquartile ranges of concentrations of plasma biomarkers per study group (**A**) Aβ40 pg/mL, (**B**) Aβ42 pg/mL, (**C**) Aβ42/40 ratio, (**D**) NfL pg/mL, (**E**) GFAP pg/mL, (**F**) p-tau181 pg/mL, (**G**) p-tau217 pg/mL. Group differences in concentrations are tested by Kruskal-Wallis tests with post-hoc comparisons using Wilcoxon tests, implementing Benjamini-Hochberg adjustments for multiple comparison. There were significant group differences in plasma Aβ42 (χ²=9.62, *p* = .009), Aβ42/40 ratio (χ²=13.26, *p* = .001), GFAP (χ²=23.04, *p* < .001), NfL (χ²=19.51, *p* < .001), p-tau181 (χ²=15.55, *p* < .001), p-tau217 (χ²=23.17, *p* < .001). Wilcoxon tests are performed to investigate which groups differ from each other, these results are displayed in the figure by significance lines (* = *p* < .05; ** = *p* < .01; *** = *p* < .001). Results are based on complete cases, there were no missing observations except for p-tau217; here one sample of an individual in the SCD group had to be excluded due to a technical issue. Abbreviations: Aβ = amyloid beta; GFAP = Glial Fibrillary Acidic Protein; NfL = neurofilament light; p-tau = phosphorylated tau; SCD = subjective cognitive decline; AD = Alzheimer’s disease.

One patient in the SCD group had an unsuccessful p-tau217 measurement due to a Simoa error, this sample was not measured again. Two samples (1 AD, 1 SCD) had analytes for p-tau217 below the LLOQ (0.15 and 0.12 pg/mL, respectively). Imputing these with the LLOQ (0.16 pg/mL) yielded similar results, hence we used the imputed values as is in line with existing literature^22^. This resulted in no missing data for the plasma analyses (Boxers, *N* = 9; SCD, *N* = 14; AD, *N* = 15), except for p-tau217 (Boxers, *N* = 9; SCD, *N* = 13; AD, *N* = 15). The average CV’s of p-tau181 and p-tau217 were 5.9% and 6.1%, respectively. Concentrations of plasma biomarkers are described in Table 1 and group differences are displayed in Fig. 1. Kruskal-Wallis tests showed significant group differences in plasma Aβ42 (χ²=9.62, *p* = .009), Aβ42/40 ratio (χ²=13.26, *p* = .001), GFAP (χ²=23.04, *p* < .001), NfL (χ²=19.51, *p* < .001), p-tau181 (χ²=15.55, *p* < .001), p-tau217 (χ²=17.30, *p* < .001). Wilcoxon tests with Benjamini Hochberg adjustments showed that boxers had higher plasma concentrations of GFAP (δ = 0.810 [CI = 0.133, 0.971, *p* < .001), p-tau181 (δ = 0.540 [CI=−0.043, 0.849], *p* = .049) and p-tau217 (δ = 0.508 [CI=−0.055, 0.826], *p* = .046)) than patients with SCD. For NfL, there was a statistical trend towards a higher concentration in boxers compared to patients with SCD (δ = 0.492 [CI = 0.003, 0.791], *p* = .053). Boxers were found to have lower plasma GFAP (δ=−0.570 [CI=−0.863, 0.007], *p* = .021), NfL (δ=−0.630 [CI=−0.887, 0.070], *p* = .015) and p-tau217 (δ=−0.698 [CI=−0.923, − 0.119], *p* = .007) than patients with AD, there was a downward trend of p-tau181 (δ=−0.437 [CI=−0.786, −0.122], *p* = .084). Aβ42 concentrations and Aβ42/40 ratios were higher in boxers compared to patients with AD (δ = 0.763 [CI = 0.307, 0.934], *p* = .004 and δ = 0.793 [0.060, 0.970], *p* = .002, respectively) and did not differ from patients with SCD (δ = 0.429 [CI=−0.053, 0.749], *p* = .134 and δ = 0.175 [CI = 0.322, 0.596], *p* = .516, respectively). Correlations between these plasma biomarkers for each group are provided in heatmaps in supplementary Figure S1.

## Area under the curve (GFAP, p-tau181, p-tau217)

The Kruskal-Walllis between-group analyses showed plasma GFAP, p-tau181 and p-tau217 to differ between boxers and patients with SCD. Analysing the area under the curve (AUC) for GFAP and p-tau of the panel when comparing boxers to patients with SCD showed GFAP to have the highest discriminative ability (AUC = 0.905; CI = 0.781–1.000) followed by p-tau181 (AUC = 0.770; CI = 0.535–1.000) and p-tau217 (AUC = 0.762; CI = 0.538–0.985). Since GFAP levels in the overall sample were shown to be influenced by age, we have performed a sensitivity where we adjusted GFAP levels for age in a regressino model, and using the residuals from this model as values for the AUC. This yielded a reduced AUC when comparing boxers to SCD (AUC = 0.786 (CI = 0.567–1.000)) and comparing boxers to AD (AUC = 0.570 (CI = 0.327–0.814)). While these AUC values are lower, isolating the discriminative effect of GFAP beyond effects of age still show ability of GFAP to differentiate between boxers and SCD, highlighting robustness of the finding.

## **Combined plasma panel (GFAP**,** p-tau181**,** p-tau217)**

GFAP and p-tau are considered to measure distinct biological processes. To investigate potential discriminative ability of a panel of biomarkers, we created z-scores (calculated within the current study sample) of GFAP, p-tau181 and p-tau217. These z-scores were averaged to create a single value representing one plasma panel score, see Figure S2. Kruskal-Wallis tests showed group differences on the plasma panel (χ²=23.45, *p* < .001). Boxers had higher plasma panel scores compared to SCD (δ = 0.778 [CI = 0.092, 0.963], *p* = .002) and lower concentrations compared to AD (δ=−0.667 [CI=−0.892, 0.177], *p* = .007). A receiver operating characteristic (ROC) analysis for the proposed panel in comparing boxers to SCD showed an AUCof 0.889 (CI = 0.756–1.000), hence lower than GFAP alone. Analysis of differentiating ability of the plasma panel between boxers and AD showed an AUC of 0.833 (CI = 0.654–1.000).

## Neuropsychological assessment

Memory composite z scores differed between the groups (χ²=18.01, *p* < .001). Post-hoc tests revealed the memory composite did not differ significantly between boxers and SCD patients (δ=−0.476 [CI=−0.791, 0.038], *p* = .062). Boxers had higher scores on memory tests than patients with AD (δ = 0.778 [CI = 0.137, 0.960], *p* = .002). EF composite z scores did not differ between the groups (χ²=3.37, *p* = .185).

### Longitudinal data for five boxers

All repeated measurements are provided in Supplementary Table S2. In the average follow-up time of 6.8 years, the rates of change of the plasma biomarkers in pg/mL for the five boxers were as follows (estimated provided for time in weeks): Aβ42: *b* = 0.0026 (*t =* 2.29, *p* = .084); Aβ42/40: *b*=−0.000002 (*t=*−0.36, *p* = .737); GFAP: *b* = 0.05 (*t =* 1.62, *p* = .182); NfL: *b* = 0.01 (*t =* 6.26, *p* = .003); p-tau181: *b* = 0.002 (*t =* 3.02, *p* = .039); p-tau217: *b* = 0.0003 (*t =* 2.18, *p* = .095). For the cognitive composite scores, we observed that all but one boxer had decreased in the memory composite over time. Similarly, we observed a decrease in the EF composite over time, with another boxer being the exception.

## MRI regional volumes

**Fig. 2:**
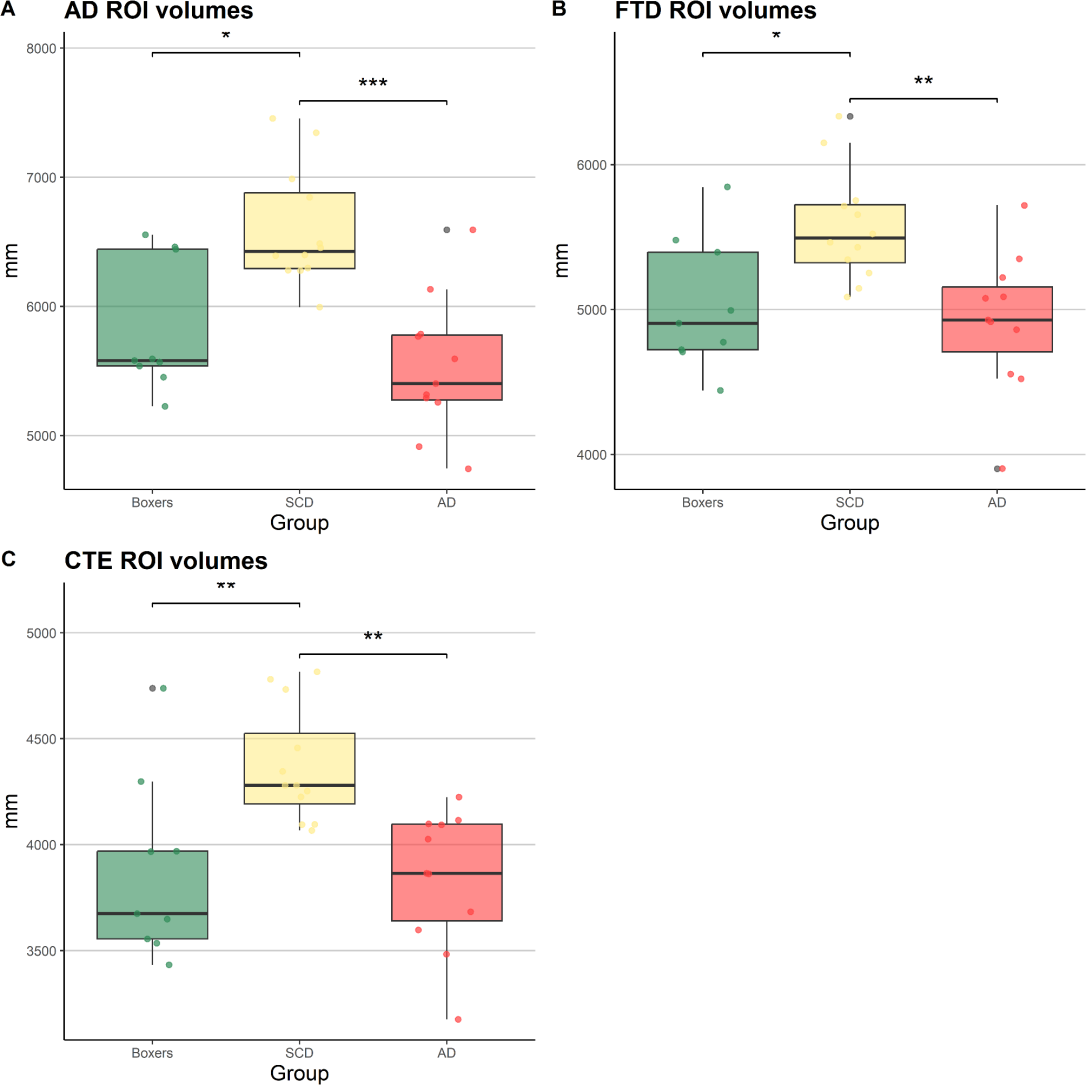
Differences in disease-related ROI volumes between groups (AD, SCD, boxers). Boxplot depicting group differences in the brain volume ROIs investigated in the current study. Group differences in volumes (mm) are tested by Kruskal-Wallis tests with Benjamini-Hochberg adjustments for multiple comparison. (**A**) AD-related = entorhinal, inferior temporal, temporal pole, inferior parietal, superior frontal, superior parietal, supramarginal, precuneus, inferior frontal, (**B**) FTD-related = insula, rostral anterior frontal, caudal anterior frontal, superior frontal, superior parietal, medial orbitofrontal, lateral orbitofrontal, (**C**) CTE-related = hippocampus, amygdala, thalamus, entorhinal, inferior parietal, superior temporal, medial orbitofrontal, lateral orbitofrontal. SCD = subjective cognitive decline, AD = Alzheimer’s disease, CTE = chronic traumatic encephalopathy, ROI = Region of Interest.

**Fig. 3:**
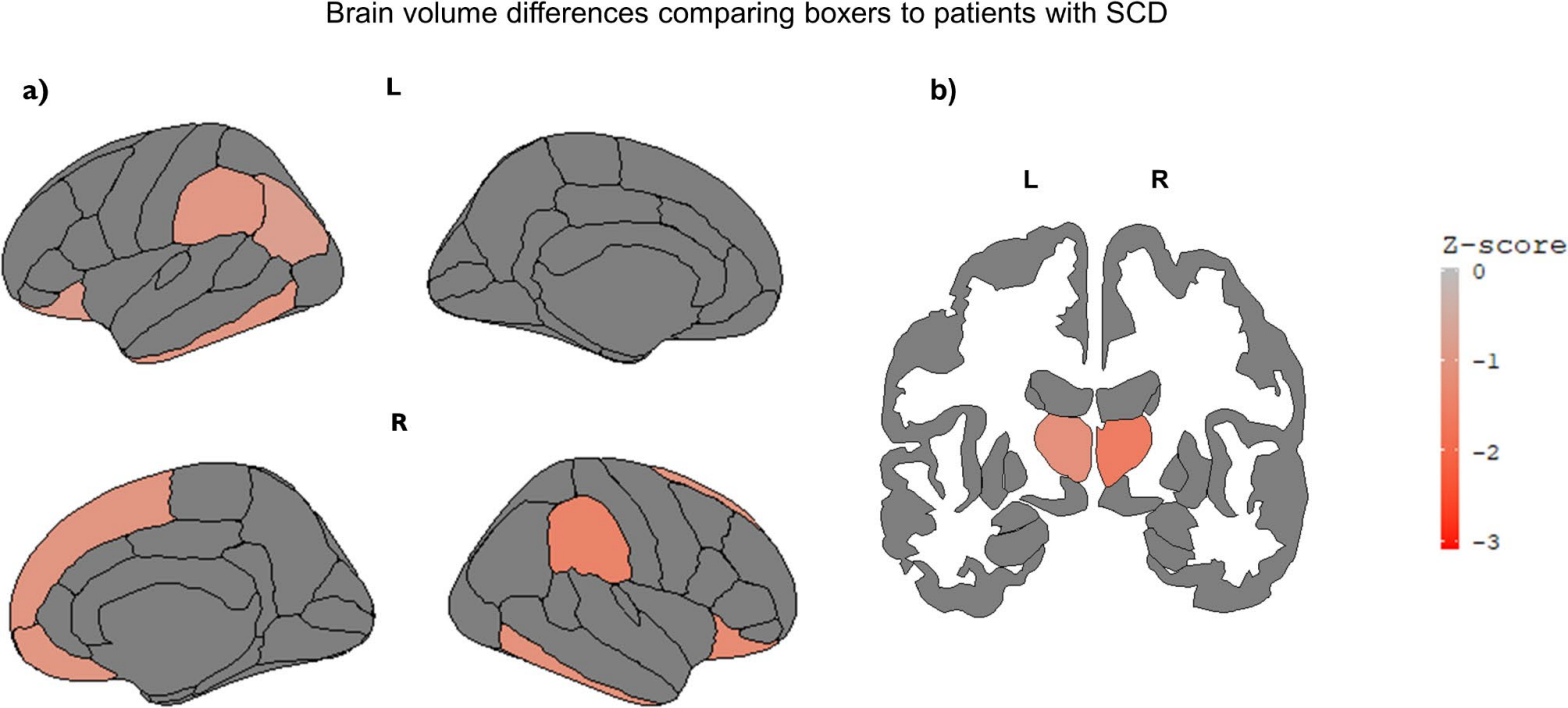
(Sub)cortical brain volume differences within the pre-specified disease-related ROIs comparing boxers to patients with SCD (**a**) cortical atlas (dk), (**b**) subcortical atlas (aseg). Brain volumes of pre-specified disease-related regions of interest (ROI’s) were derived from Freesurfer and values were z-transformed. Regions were based on the Desikan-Killiany (DK) atlas. Differences in the z-transformed volumetric values for the regions between groups were tested with t-tests. All p-values are adjusted to correct for multiple testing and the False Discovery Rate (FDR). Z-scores are indicated by the heat map on the right. The legend contains only negative Z-scores, this is due to the finding that boxers had only significantly lower volumes than patients with SCD, and not higher. The brain maps are plotted with the ggseg package in R. Regions that differed between boxers and patients with SCD, i.e. regions that showed significantly different volumes in boxers compared to patients with SCD, are colored in the brain maps. Darker colors indicate stronger differences between groups. Non-significant values are not displayed. Significant group differences were found for the following regions: lateral orbitofrontal (bilateral), inferior temporal (bilateral), supramarginal (bilateral), thalamus (bilateral), medial orbitofrontal (right), superior frontal (right), inferior parietal (left). SCD = subjective cognitive decline, L = left, R = right.

For six out of nine boxers a CSP was observed (66.67%), compared to four patients with SCD (28.57%) and six patients with AD (40%). Group differences were found in brain volumes within the pre-specified AD-related (χ²=12.99, *p* = .002), FTD-related (χ²=9.91, *p* = .007) and CTE-related (χ²=13.20, *p* = .001, Fig. 2) regions. Post-hoc tests showed that boxers had lower AD-related (δ=−0.636 [CI=−0.888, − 0.092], *p* = .028), FTD-related (δ=−0.616 [CI=−0.884, 0.042], *p* = .030) and CTE-related (δ=−0.697 [CI=−0.926, − 0.090], *p* = .011) volumes compared to patients with SCD. Differences in cortical and subcortical volumes, as determined by t-tests, between groups are plotted in Fig. 3 and Supplementary Fig. S3. No significant differences in volumes were found on the disease-related regions between boxers and patients with AD (all *p* > .05).

## Sensitivity analyses

To account for potential influences of age, BMI, and education, ranked linear models were used as an alternative to the Kruskal-Wallis test, with all plasma biomarkers (Aβ42, Aβ42/40 ratio, GFAP, NfL, p-tau181, p-tau217) and disease-related regional MRI volumes (AD-, FTD-, CTE ROIs) as outcomes. All group effects remained significant. Pairwise comparisons with BH adjustments showed the same groups to differ significantly from each other as in the original Kruskal-Wallis tests with Wilcoxon pairwise comparisons. Additionally, boxers had significantly higher concentrations of NfL compared to patients with SCD (*p* = .002; originally *p* = .053). After bootstrapping, in the comparisons between boxers and the SCD group, the p values of the main analyses remain robust for GFAP (*p* < .001), and not for p-tau181 (*p* = .129) and p-tau217 (*p* = .129). The comparisons in AD- (*p* = .087), FTD- (*p* = .108) or CTE-related (*p* = .069). ROI volumes between boxers and SCD also lose significance after bootstrapping.

### Relations between multimodal biomarkers

Among the boxers, linear regression analyses showed no significant associations between any of the plasma biomarkers and the AD-, FTD-, or CTE related regional volumes, neither with neuropsychological domain composite z scores of memory and EF. In addition no significant relations between the combined plasma panel on the AD-related (*b=*−287.0; *p* = .535), FTD-related (*b=*−320.0; *p* = .430) and CTE-related (*b*=−167.2; *p* = .667) volumes were detected. In these explorative analyses, plotting the non-significant regressions between plasma GFAP (the marker with best differentiating ability in the current study) and the disease-related regional volumes revealed modest negative relations, as shown in Supplementary Figure S4.

## Discussion

Our results showed higher plasma GFAP, p-tau181 and p-tau217 in former boxers compared to patients with SCD. Boxers had lower plasma concentrations compared to patients with AD for all plasma biomarkers, except for plasma Aβ42 and Aβ42/40. In subsequent explorative analyses, GFAP showed the best differentiating ability between boxers and patients with SCD. Boxers had lower volumes in pre-specified disease-related brain regions (composed for AD, FTD and CTE) than patients with SCD, but not compared to patients with AD. The current findings suggest that the former boxers showed biological processes indicative for neuronal damage, which may put them at risk for neurodegenerative disease, though the underlying etiology remains unclear.

The finding that GFAP was elevated in boxers compared to patients with SCD is supported by previous research^15,19^. Higher levels of plasma GFAP in boxers suggest elevated astrocyte activation following RHI in boxing. Whilst astrocytes are important in maintaining integrity of the blood-brain barrier, the ongoing elevation can contribute to neuroinflammation and neurodegeneration, which are processes associated with i.e. CTE^23,24^. Plasma NfL was higher in boxers compared to patients with SCD on a trend level. Earlier studies found more convincing elevations in NfL^9,13^. Although the direction of the trend found in the current study did corroborate to these previous studies, the lack of significance was possibly related to lack of power due to the small sample size.

Concentrations of plasma Aβ42 and Aβ42/40 were highest among boxers, which indicated that among the three groups under investigation boxers had the least pathogenic concentrations. The role of Aβ pathology in CTE has been disputed, since previous literature suggests that Aβ neuritic plaques are not a hallmark of the disease^3,4^while amyloid has also been suggested to be a driving factor of tau phosphorylation in CTE^12^. P-tau181 and p-tau217 were higher in boxers compared to patients with SCD. This result is supported by earlier findings regarding retired contact sports players^12,25^. The current results suggest elevation of phosphorylated tau without indication of elevated amyloid in the brain. Since for ‘typical’ AD we can expect a decreased plasma Aβ42/40 ratio and elevated p-tau181 and p-tau217^26^, observing elevated p-tau in RHI-exposed individuals without a decreased Aβ42/40 ratio might hint more towards underlying CTE-pathology than AD-pathology only. However, this should be interpreted with caution because the majority of boxers did not have amyloid biomarkers measured with CSF or amyloid PET, which are the current gold standards. In addition, earlier studies reported p-tau levels of two football players and one soccer player with post-mortem confirmed CTE pathology not being significantly elevated^12,27^. Furthermore, it was found that p-tau181 and p-tau217 were not significantly elevated in PET-amyloid negative TES individuals^12^. Along with the results of the current study, these findings highlight the need for further investigation of the interaction between amyloid and tau in RHI-exposed individuals.

We observed elevations of the plasma biomarkers within the boxers over a seven year time follow-up, except for the Aβ42/40 ratio, which decreased. The elevations of NfL and p-tau181 were statistically significant, suggesting potential increases in indicators for axonal injury and tau pathology. Elevations in tau among retired fighters has been described in earlier work^9^. Heterogeneity in the findings of which biomarkers elevate over time underscores the complexity of the interplay and dynamics of biomarkers over time. While the findings are not generalizable due to the small sample size, they are exploratively interesting as they may reflect underlying processes in individuals at risk for neurodegeneration. Since most boxers declined in their neuropsychological performance and progressed in their TES classification, it is apparent that there is some progression in cognitive dysfunction that could be partly related to the increased plasma biomarker concentrations. Future longitudinal studies with larger samples could provide more definitive insights into how biomarker trajectories relate to cognitive decline and disease progression.

Our results regarding the brain volumes suggest that the former professional boxers had lower volumes in AD-, FTD- and CTE-related regions compared to patients with SCD. Further inspection of the individual areas within the pre-specified disease-related regions showed that boxers had lower volumes than patients with SCD in regions including the bilateral thalamus and orbitofrontal cortices (Fig. 3). Accordingly, earlier studies have reported these regions to be affected by pathological tau in patients with CTE^25^or to be smaller after RHI exposure^16^, indicating these regions as being potentially vulnerable for atrophy after RHI. Whilst boxers did not differ from patients with AD on the composed ROIs, inspection of individual areas within the pre-specified ROIs showed patients with AD had lower volumes on regions including the bilateral precuneus and isthmus cingulate cortices (Supplementary Figure S3).

In the current study, our unique sample consisted of boxers with a well-documented history of repetitive head impacts (RHI) with a detailed overview of plasma and imaging data. The current study also has a few limitations. First, the sample size of boxers was small, which led to reduced statistical power of the analyses and limited the potential to adjust for potential confounders. We aimed to mitigate this by performing sensitivity analyses, linear ranked models controlled for covariates and bootstrapping of the results showed similar outcomes, adding robustness to the findings. Second, the sample in this study included only males, thus making the results not generalizable to females. Third, there were some demographic differences between groups. Boxers had on average lower education than patients with SCD. Since boxers were not found to differ in cognitive performance overall from patients with SCD, it is not expected that factors as educative background could fully explain differences in our outcome variables. In addition, no differences between boxers and patients with SCD in other known risk factors such as cardiovascular risk or depressive symptoms were observed. Further, patients with AD were on average seven years older than the boxers. Nevertheless, this still provides a very young cohort of patients with AD, and sensitivity analyses where age is added as a covariate shows no significant influence of age on the reported results. Fourth, there was some heterogeneity within the boxer sample with regards to their ages and careers (i.e. time since the last competition, years of exposure). This may have introduced variability in the data with regards to the onset of long term outcomes of exposure. However, the common and defining element in the boxer group is the extensive exposure to head impacts, thereby allowing them to be grouped as being at risk for CTE^28^. Nevertheless, future larger studies should explore potential temporal dynamics of biomarkers by stratifying on age or time since last exposure, to investigate performance of biomarkers in the context of acute vs. non-acute processes. Fifth, different MRI scanners were used for the baseline MRI assessments. Harmonization was not feasible due to the small numbers, therefore there may be some variability in MRI data due to scanner type. Lastly, the current study lacks neuropathological data. Although the results seem to reflect potential neuronal damage in the boxers, we can only evaluate if there is risk for neurodegeneration and can make no inferences regarding the etiology, i.e. CTE or AD.

Additional research is needed to study plasma biomarkers concentrations in a larger cohort of boxers. Future studies should focus on including female boxers, since they are largely underrepresented in current literature. There would also be great scientific value in adding post mortem data in future studies to correlate in vivo biomarkers with neuropathological data. The findings on MRI indicate specific patterns of brain atrophy in regions vulnerable to RHI-related injury (e.g., thalamus and orbitofrontal cortices). Future studies could explore whether combining imaging biomarkers with plasma biomarkers enhances the ability to differentiate between RHI-related neurodegeneration, AD, and healthy aging, especially in the preclinical stages. Further, in the current study we investigated validated biomarkers for AD pathology and neuronal damage, but future studies should also include other biomarkers that could be more specific to RHI-related neurodegeneration. For example, subtle changes in phosphorylated tau may be captured by measuring the ratio of p-tau to total tau^29^. Further, the novel biomarker brain-derived tau has differential ability between AD and other neurodegenerative diseases and has yet to be investigated in the context of RHI and CTE^30^. In the current study, two of the boxers were carriers of the APOE-E4 allele. Since in both CTE and AD carriership of the E4 allele is known to be related to disease progression, future studies should investigate the role of the APOE genotype in RHI-related neurodegeneration further^31^. As of the start of 2025 the five boxers still participate actively in the NEwTON cohort study, meaning they have completed or will complete a final follow-up visit with plasma, MR imaging and cognition. This allows for more extensive longitudinal assessment in the future, as well as the ability to monitor possible progression of neurodegenerative processes.

## Conclusion

In summary, plasma GFAP, p-tau181 and p-tau217 were higher in boxers than in patients with SCD, whilst boxers had lower brain MRI volumes in pre-specified disease-related ROIs, indicating underlying biological evidence of neurodegenerative processes in the boxers. Boxers differed from AD by having higher plasma Aβ42 and Aβ42/40, lower NfL, GFAP, p-tau181 and p-tau217, whilst on neuroimaging no differences in brain atrophy were found. Plasma GFAP, p-tau181 and p-tau217 as well as regional brain atrophy may be useful biomarkers for neurodegenerative risk following RHI. The results of the current study point to a compelling need to further characterize performance of plasma biomarkers and atrophy patterns in individuals exposed to RHI to improve timely recognition of neurodegenerative risk. Validating potential biomarkers like GFAP and integrating these with imaging biomarkers in larger, more diverse populations will allow for clinical applications, such as routine screening of athletes or individuals with a history of RHI. Early identification of neurodegenerative risk could inform preventative strategies or targeted interventions.

## Methods

### Boxers sample

Nine male former professional boxers (age range: 46–70 years (*M* = 55.7; *SD =*7.7) had visited the memory clinic of Amsterdam UMC on invitation, as part of a pilot study for the consequences of boxing. Measurements were performed 6–30 years after the last professional competition. Five of these boxers had follow-up measurements as part of the NEwTON study, a prospective cohort study including individuals at risk for CTE^32^. The follow-up duration of the five boxers ranged from 6 to 7 years with a mean of 6.8 years (Age range during follow-up 53–77 years; *M* = 62.0; *SD*= 8.9). One boxer was classified as having ADHD (did not use medication) and one boxer had recovered from a benzodiazepine addiction. There was no relevant medical history for the other boxers. Three of the boxers underwent a lumbar puncture, with CSF Aβ levels assessed using ELISA (Innotest, abnormal < 550 pg/mL)^33^. None underwent an amyloid Positron Emission Tomography (PET) scan. After the first assessment, boxers were classified in a multidisciplinary meeting according to the NIA-AA diagnostic criteria for AD^34^. The boxers were classified by a multidisciplinary study team for the Traumatic Encephalopathy Syndrome (TES) according to the 2021 NINDS TES criteria^28^. Detailed classifications are provided in the results section.

### Boxing careers

Eight out of nine boxers had a professional career in boxing. The other boxer did not compete at the professional level, but did participate in many competitions at amateur level for more than a decade. Three boxers had also participated in recreational soccer. Six boxers regularly wore head protection in training, only one boxer had used head protection during competition. A public record for boxing statistics was consulted for information regarding the professional careers^35^. This information is not (publicly) available for amateur boxing, so for the amateur boxer, we relied on self-reported information. Only one out of nine boxers reported at least one previous concussion, using a standardized questionnaire stating: “Have you ever suffered from a concussion”. Because the number of knock-outs conflicted with the self-reported number of concussions, concussion history was most likely underreported and was not considered further.

### Comparison sample (Amsterdam dementia Cohort)

Comparison groups of 30 male patients matched on age and sex without any history of traumatic brain injury (TBI) or contact sports were drawn from the Amsterdam Dementia Cohort (ADC)^36^. The comparison sample consisted of 15 patients with dementia due to AD that were amyloid positive and 14 patients with Subjective Cognitive Decline (SCD) as healthy controls that were amyloid negative as determined by CSF and had no cognitive impairment based on extensive cognitive testing. We chose patients with AD and SCD for the comparison sample, since these patients have a biomarker supported diagnosis and allow for comparison with patients with and without neurodegenerative disease. Furthermore, recent literature describes potential discriminative ability of plasma biomarkers between AD and other neurodegenerative diseases^37^. Criteria for selecting the subjects were an age between 46 and 70 years, male sex, and availability of ethylenediaminetetraacetic acid (EDTA) plasma within the biobank of the ADC. Patients were only selected if their first and last known diagnoses in the ADC were either AD or SCD, to minimize the chance that patients had converted to another diagnosis during clinical follow-ups. Contact sport and head trauma history were assessed by a standardized questionnaire, including questions about any life-time history of sport participation as well as concussion history. This was supported by comprehensive screening of medical records to obtain additional information on any head injury incidence. Patients were excluded from selection if there was any mention of contact sports, head trauma, or concussion. Similarly, patients were excluded if a history of stroke or kidney disease was reported in the their medical record since these factors are known to potentially influence blood plasma levels^38,39^. One patient record within the comparison sample included a reference to a potential history of fighting. This patient was contacted by telephone and specified that he participated in recreational judo and never experienced head impacts, therefore this patient was not excluded. Four individuals with AD were prescribed galantamine. There were 11 subjects that used medication for cardiovascular symptoms such as hypertension.

### Plasma biomarkers

APOE genotype was determined during baseline visits. Nonfasted blood was collected during baseline measurements through intravenous phlebotomy in EDTA tubes, and then centrifuged so plasma could be taken. Aliquots of 0.5 mL were stored at −80 °C within 60 min of sample collection. For analysis, aliquots from left-over plasma were selected that had no prior freeze-thaw cycles. All analyses were performed at the Neurochemistry Laboratory, Amsterdam UMC, VUmc, Amsterdam, the Netherlands. Analyses of plasma was performed according to manufacturer’s instructions. Concentrations of Aβ40, Aβ42, GFAP and NfL were measured in monoplo with the commercial Neurology 4-plex E kit (catalog no. 103670) using single molecule array technology (Simoa; Quanterix, Billerica, MA, USA). The CV% stability in plasma samples of this kit, has consistently been shown to be, on average, below 7% in our lab, allowing for the measurements to be run in monoplo. P-tau181 and p-tau217 were measured in duplicate using Simoa pTau181 V2 Advantage kit (Quanterix, catalog no. 103714) and p-tau217 assay (ALZpath, Carlsbad, CA, USA, no official catalog no.; ADIP220365), respectively. Before conducting the analysis, samples were thawed at room temperature, followed by thorough mixing and centrifugation at 10,000 x*g*for 10 min. Calibrators were thawed at room temperature and stored on ice with the reagents until the measurements were initiated. In a single run, plasma samples were loaded into a Quanterix 96-well plate, this run included lot-specific reagents along with two quality controls (one low and one high). The Lower Limits of Qualification (LLOQ) in EDTA plasma were as follows: Aβ40 = 4.08 pg/mL, Aβ42 = 1.51 pg/mL, NfL = 1.6 pg/mL, GFAP = 11.6 pg/mL, p-tau181 = 0.338 pg/mL, p-tau217 = 0.16 pg/mL. If measurements would be below the LLOQ, they are imputed with the LLOQ value as recommended in previous literature^40,41^The cut-off used for the coefficients of variance (CV) of the samples run in duplicate was 20%^42,43^.

### Imaging

3D MRI brain images were acquired. The field strength was 1.5 or 3.0. All follow up scans were done with a GE Signa 3T scanner. Coronal and axial sections of the brain MRIs were reviewed by a trained researcher (SK) to establish visibility of a CSP. Presence of a CSP was scored dichotomous (yes/no) based on visibility of a fluid-filled cavity within the septum pellucidum^44^. MRI scans were processed using FreeSurfer V6.0. Due to variable availability within the ADC for Freesurfer segmented data, for the imaging analyses the sample consisted of 11 patients with AD, 11 patients with SCD and 9 boxers. Anatomical regions were defined with the Desikan-Killiany (DK) atlas implemented in Freesurfer^45^. Acquired volumetric values were all controlled for total intracranial volumes. To explore potential differences in (sub)cortical volumes between the study groups, regions of interest (ROIs) were determined based on specific patterns of neurodegenerative disease. We chose to compose ROIs for AD, frontotemporal dementia (FTD) and CTE. CTE can resemble AD and FTD on clinical characteristics, and there is no golden standard in terms of a CTE-related ROI. Region composites were established as follows: to reflect brain regions typically affected in AD^46^the AD-related composite included the bilateral entorhinal, inferior temporal, temporal poles, inferior parietal, superior frontal, superior parietal, supramarginal, precunues and caudal middle frontal cortices. For regions typically affected in FTD, based on literature^47,48^a related ROI was composed of the bilateral insula, rostral anterior cingulate, caudal anterior cingulate, superior frontal, superior parietal and lateral and medial orbitofrontal cortices. Based on current literature where neuropathological tissue with definitive CTE has been examined, certain regions are known to contain pathological tau depositions related to CTE^25^. Regions that made up the bilateral CTE-related ROI were the hippocampus, amygdala, thalamus, entorhinal, inferior parietal, superior temporal, and the medial and lateral orbitofrontal cortices. MRI volumetric data was not assessed longitudinally within the boxers. There was variability in MRI scanner used during the first visits and another MRI scanner was used during the follow-up visit. The boxer sample that had a follow-up was too small to correct for scanner and age effects, making the longitudinal MRI data not appropriate for statistical analysis.

### Neuropsychological assessment

To assess global cognitive functioning the MMSE was used. All patients underwent standard neuropsychological assessment at the time of their memory clinic visit. This 60 min assessment was done by experienced neuropsychologists and captured multiple cognitive domains, including memory, attention, processing speed, executive function, language, and visuospatial function. In the current study, we focused on memory and executive functioning (EF), as these domains constitute the core cognitive criteria of TES^49^. The memory domain composite consisted of z scores of the immediate and delayed recall of the Rey Auditory Verbal Learning Task (RAVLT) and the Visual Association Test (VAT, version A). The EF domain score was composed of the Digit span Backwards score and the total score of three trials of the Letter fluency test. Scores for each test are provided in Supplementary Table **S1**. Z scores were calculated using mean scores on each test for 440 cognitively unimpaired individuals from the ADC cohort (RAVLT total score: M = 42.4; SD = 9.0, RAVLT delayed recall: M = 8.7; SD = 2.8, Digit span backwards: M = 9.3;SD = 2.7, Letter fluency total score: M = 37.7; SD = 11.9, VAT total score: M = 11.7;SD = 0.8)^36^.

### Neuropsychiatric symptoms

Neuropsychiatric symptoms were assessed by the Neuropsychiatric Inventory (NPI), a semi-structured interview with an informant of the patient. Neuropsychiatric data was available for 14 patients with AD, 9 patients with SCD, and 3 boxers. Due to the limited availability, the NPI was not further used as an outcome measure, but only for descriptive purposes.

### Statistical analyses

All statistical analyses and data visualizations were performed using R software version 4.0.3^36^. Group differences on descriptive variables were compared using chi-square tests for categorical variables and one-way Analysis of Variance (ANOVA) tests for continuous variables. In case of significant group differences, post-hoc Tukey honest significant difference tests (HSD) were performed. Since the study samples were small and some of the outcome variables (p-tau181, p-tau21) did not follow a Gaussian distribution, non-parametric tests were used for the analyses. Differences in plasma concentrations, MRI volumetric measures and neuropsychological scores between groups (boxers vs. AD vs. SCD) at baseline were measured through Kruskal-Wallis tests. For post-hoc analyses, Wilcoxon’s test with Benjamini-Hochberg (BH) adjustment for multiple comparison was used to control the False Discovery Rate (FDR). Effect sizes of the pairwise Wilcoxon’s tests were calculated with Cliff’s Delta (R package ‘effsize’). Cliff’s Delta is appropriate for non-normal distributions and ranges from − 1 to 1, allowing the values to be interpreted as small effects (around δ = 0.147), medium effects (δ = 0.33) and large effects (δ = 0.474)^50^. Because these tests do not allow for adding covariates, we perform sensitivity analyses where age, BMI and education (Verhage) are added as covariates. First, we perform ranked linear models for each of the plasma and imaging outcomes. With the estimated marginal means we perform pairwise comparisons with BH adjustments for multiple comparison. Second, to mitigate the small sample size, we employed bootstrapping as a resampling method for the Kruskal-Wallis tests. We resampled the data 1000 times to generate a distribution of p-values, which again were adjusted with the BH method. Receiver Operating Characteristic (ROC) curves were performed to generate the Area under the Curve (AUC) values with their respective Confidence Intervals (CIs). In these analyses, a CI that did not contain zero was considered statistically significant. We performed linear regression analyses to test associations between plasma biomarkers and outcomes on MRI volumetrics and neuropsychological performance, as well as linear regressions to control for effects of age on the plasma biomarker concentrations. Due to the small sample size, ROC and regression analyses are performed exploratively. Visualization of z-scaled (sub)cortical volumetric differences (as measured with t-tests) between groups was done with the ggseg package using the Desikan-Killiany brain atlas^45^. To assess longitudinal changes within boxers, we describe the rate of change in concentrations of Aβ42, the Aβ40/Aβ42 ratio, p-tau181, p-tau217, GFAP and NfL from their first visits to the follow-up measurement during the NEwTON visit. Linear mixed-effects models (LMMs) were used to exploratively examine effects of time (in weeks) on the plasma biomarkers, due to the small sample with longitudinal data these results should be interpreted with caution as they could not be corrected for covariates. For the LMMs we used the lme4 package^37^. Since only five boxers had a follow-up measurement, longitudinal measurements were interpreted qualitatively. A p-value lower than 0.05 was deemed statistically significant and CIs were set at 95%.

## Electronic supplementary material

Below is the link to the electronic supplementary material.

Supplementary Material 1

## Data Availability

The datasets used and analyzed during the current study are available from the corresponding author on reasonable request.

## Abbreviations

AD: Alzheimer disease
SCD: Subjective cognitive decline
FTD: Frontotemporal dementia
RHI: Repetitive head injury
TBI: Traumatic brain injury
Aβ: Amyloid-beta
NfL: Neurofilament Light
GFAP: Glial Fibrillary Protein
p-tau: Phosphorylated tau
CDR: Clinical Dementia Rating
CTE: Chronic traumatic encephalopathy
CV: Coefficient of variation
LLOQ: Lower limit of quantification
MCI: Mild cognitive impairment
TES: Traumatic encephalopathy syndrome
ROI: Region of interest
MRI: Magnetic resonance imaging
CSP: Cavum septum pellucidum

## References

[1] Gallacher, J. et al. Amateur boxing and dementia: cognitive impairment within the 35-Year caerphilly cohort study. *Clin. J. Sport Med.* **32** (3), 329–333 (2022). 35470341 10.1097/JSM.0000000000000976

[2] Shahim, P. et al. Fluid biomarkers for chronic traumatic encephalopathy. *Semin Neurol.* **40** (4), 411–419 (2020). 32740901 10.1055/s-0040-1715095

[3] McKee, A. C. et al. The spectrum of disease in chronic traumatic encephalopathy. *Brain* **136** (Pt 1), 43–64 (2013). 23208308 10.1093/brain/aws307 PMC3624697

[4] Bieniek, K. F. et al. The second NINDS/NIBIB consensus meeting to define neuropathological criteria for the diagnosis of chronic traumatic encephalopathy. *J. Neuropathol. Exp. Neurol.* **80** (3), 210–219 (2021). 33611507 10.1093/jnen/nlab001 PMC7899277

[5] MARTLAND, H. S. *PUNCH DRUNK J. Am. Med. Association*, **91**(15): 1103–1107. (1928).

[6] Franz, G. et al. Amyloid beta 1–42 and Tau in cerebrospinal fluid after severe traumatic brain injury. *Neurology* **60** (9), 1457–1461 (2003). 12743231 10.1212/01.wnl.0000063313.57292.00

[7] Neselius, S. et al. Olympic boxing is associated with elevated levels of the neuronal protein Tau in plasma. *Brain Inj*. **27** (4), 425–433 (2013). 23473386 10.3109/02699052.2012.750752

[8] Gill, J. et al. Higher Exosomal Tau, amyloid-beta 42 and IL-10 are associated with mild TBIs and chronic symptoms in military personnel. *Brain Inj*. **32** (10), 1277–1284 (2018). 29913077 10.1080/02699052.2018.1471738 PMC6129391

[9] Bernick, C. et al. Longitudinal performance of plasma neurofilament light and Tau in professional fighters: the professional fighters brain health study. *J. Neurotrauma*. **35** (20), 2351–2356 (2018). 29609512 10.1089/neu.2017.5553

[10] Oliver, J. M. et al. Fluctuations in blood biomarkers of head trauma in NCAA football athletes over the course of a season. *J. Neurosurg.* 1–8 (2018). 10.3171/2017.12.JNS172035 29807487

[11] Vasilevskaya, A. et al. Investigating the use of plasma pTau181 in retired contact sports athletes. *J. Neurol.* **269** (10), 5582–5595 (2022). 35751688 10.1007/s00415-022-11223-7

[12] Asken, B. M. et al. Plasma P-tau181 and P-tau217 in patients with traumatic encephalopathy syndrome with and without evidence of alzheimer disease pathology. *Neurology* **99** (6), e594–e604 (2022). 35577574 10.1212/WNL.0000000000200678 PMC9442622

[13] Hiskens, M. I. et al. Blood biomarkers for assessment of mild traumatic brain injury and chronic traumatic encephalopathy. *Biomarkers* **25** (3), 213–227 (2020). 32096416 10.1080/1354750X.2020.1735521

[14] Stein, T. D. et al. Beta-amyloid deposition in chronic traumatic encephalopathy. *Acta Neuropathol.* **130** (1), 21–34 (2015). 25943889 10.1007/s00401-015-1435-y PMC4529056

[15] Zetterberg, H. et al. Neurochemical aftermath of amateur boxing. *Arch. Neurol.* **63** (9), 1277–1280 (2006). 16966505 10.1001/archneur.63.9.1277

[16] Bernick, C. et al. Repeated head trauma is associated with smaller thalamic volumes and slower processing speed: the professional fighters’ brain health study. *Br. J. Sports Med.* **49** (15), 1007–1011 (2015). 25633832 10.1136/bjsports-2014-093877 PMC4518758

[17] Alosco, M. L. et al. Structural MRI profiles and Tau correlates of atrophy in autopsy-confirmed CTE. *Alzheimers Res. Ther.* **13** (1), 193 (2021). 34876229 10.1186/s13195-021-00928-y PMC8653514

[18] Asken, B. M. & Rabinovici, G. D. Identifying degenerative effects of repetitive head trauma with neuroimaging: a clinically-oriented review. *Acta Neuropathol. Commun.* **9** (1), 96 (2021). 34022959 10.1186/s40478-021-01197-4 PMC8141132

[19] Bernick, C. et al. Blood biomarkers and neurodegeneration in individuals exposed to repetitive head impacts. *Alzheimers Res. Ther.* **15** (1), 173 (2023). 37828595 10.1186/s13195-023-01310-w PMC10571311

[20] Swann, O. J. et al. Fluid biomarkers and risk of neurodegenerative disease in retired athletes with multiple concussions: results from the international concussion and head injury research foundation brain health in retired athletes study of ageing and Impact-Related neurodegenerative disease (ICHIRF-BRAIN study). *BMJ Open. Sport Exerc. Med.* **8** (3), e001327 (2022). 36111130 10.1136/bmjsem-2022-001327 PMC9438045

[21] Asken, B. M. et al. *Alzheimer’s Pathology Is Associated With Altered Cognition, Brain Volume, and Plasma Biomarker Patterns in Traumatic Encephalopathy Syndrome*15p. 126 (Alzheimer’s Research & Therapy, 2023). 1. 10.1186/s13195-023-01275-w PMC10360257 37480088

[22] Kritikos, M. et al. Plasma amyloid beta 40/42, phosphorylated Tau 181, and neurofilament light are associated with cognitive impairment and neuropathological changes among world trade center responders: A prospective cohort study of exposures and cognitive aging at midlife. *Alzheimers Dement. (Amst)*. **15** (1), e12409 (2023). 36911360 10.1002/dad2.12409 PMC9994167

[23] McKee, A. C. et al. The first NINDS/NIBIB consensus meeting to define neuropathological criteria for the diagnosis of chronic traumatic encephalopathy. *Acta Neuropathol.* **131** (1), 75–86 (2016). 26667418 10.1007/s00401-015-1515-z PMC4698281

[24] Shahim, P. et al. Blood biomarkers for brain injury in concussed professional ice hockey players. *JAMA Neurol.* **71** (6), 684–692 (2014). 24627036 10.1001/jamaneurol.2014.367

[25] Alosco, M. L. et al. Associations between near end-of-life flortaucipir PET and postmortem CTE-related Tau neuropathology in six former American football players. *Eur. J. Nucl. Med. Mol. Imaging*. **50** (2), 435–452 (2023). 36152064 10.1007/s00259-022-05963-x PMC9816291

[26] Brickman, A. M. et al. Plasma p-tau181, p-tau217, and other blood-based Alzheimer’s disease biomarkers in a multi-ethnic, community study. *Alzheimers Dement.* **17** (8), 1353–1364 (2021). 33580742 10.1002/alz.12301 PMC8451860

[27] van Amerongen, S. et al. Severe CTE and TDP-43 pathology in a former professional soccer player with dementia: a clinicopathological case report and review of the literature. *Acta Neuropathol. Commun.* **11** (1), 77 (2023). 37161501 10.1186/s40478-023-01572-3 PMC10169296

[28] Katz, D. I. et al. National Institute of neurological disorders and stroke consensus diagnostic criteria for traumatic encephalopathy syndrome. *Neurology* **96** (18), 848–863 (2021). 33722990 10.1212/WNL.0000000000011850 PMC8166432

[29] Kyalu Ngoie Zola, N. et al. Specific post-translational modifications of soluble Tau protein distinguishes Alzheimer’s disease and primary Tauopathies. *Nat. Commun.* **14** (1), 3706 (2023). 37349319 10.1038/s41467-023-39328-1 PMC10287718

[30] Halicki, M. J., Hind, K. & Chazot, P. L. Blood-Based Biomarkers in the Diagnosis of Chronic Traumatic Encephalopathy: Research to Date and Future Directions. *Int. J. Mol. Sci.* **24**(16), (2023). 10.3390/ijms241612556 PMC10454393 37628736

[31] Atherton, K. et al. Association of APOE genotypes and chronic traumatic encephalopathy. *JAMA Neurol.* **79** (8), 787–796 (2022). 35759276 10.1001/jamaneurol.2022.1634 PMC9237800

[32] van Amerongen, S. et al. Rationale and design of the neurodegeneration: traumatic brain injury as origin of the neuropathology (NEwTON) study: a prospective cohort study of individuals at risk for chronic traumatic encephalopathy. *Alzheimers Res. Ther.* **14** (1), 119 (2022). 36050790 10.1186/s13195-022-01059-8 PMC9438060

[33] Mulder, C. et al. Amyloid-beta(1–42), total Tau, and phosphorylated Tau as cerebrospinal fluid biomarkers for the diagnosis of alzheimer disease. *Clin. Chem.* **56** (2), 248–253 (2010). 19833838 10.1373/clinchem.2009.130518

[34] McKhann, G. M. et al. The diagnosis of dementia due to Alzheimer’s disease: recommendations from the National Institute on Aging-Alzheimer’s association workgroups on diagnostic guidelines for Alzheimer’s disease. *Alzheimers Dement.* **7** (3), 263–269 (2011). 21514250 10.1016/j.jalz.2011.03.005 PMC3312024

[35] *BoxRec*. (2023).

[36] van der Flier, W. M. & Scheltens, P. Amsterdam dementia cohort: performing research to optimize care. *J. Alzheimers Dis.* **62** (3), 1091–1111 (2018). 29562540 10.3233/JAD-170850 PMC5870023

[37] Teunissen, C. E. et al. Blood-based biomarkers for Alzheimer’s disease: towards clinical implementation. *Lancet Neurol.* **21** (1), 66–77 (2022). 34838239 10.1016/S1474-4422(21)00361-6

[38] Chen, J. et al. Reference intervals for plasma amyloid-β, total Tau, and phosphorylated tau181 in healthy elderly Chinese individuals without cognitive impairment. *Alzheimers Res. Ther.* **15** (1), 100 (2023). 37237388 10.1186/s13195-023-01246-1 PMC10214719

[39] Berry, K. et al. Hepatic and renal function impact concentrations of plasma biomarkers of neuropathology. *Alzheimers Dement. (Amst)*. **14** (1), e12321 (2022). 35845260 10.1002/dad2.12321 PMC9274803

[40] Lee, M., Kong, L. & Weissfeld, L. Multiple imputation for left-censored biomarker data based on Gibbs sampling method. *Stat. Med.* **31** (17), 1838–1848 (2012). 22359320 10.1002/sim.4503

[41] Herbers, J. et al. How to deal with non-detectable and outlying values in biomarker research: best practices and recommendations for univariate imputation approaches. *Compr. Psychoneuroendocrinol*. **7**, 100052 (2021). 35757062 10.1016/j.cpnec.2021.100052 PMC9216349

[42] Beal, S. L. Ways to fit a PK model with some data below the quantification limit. *J. Pharmacokinet. Pharmacodyn*. **28** (5), 481–504 (2001). 11768292 10.1023/a:1012299115260

[43] Tholen, D. W. et al. Protocols for determination of limits of detection and limits of quantitation; approved guideline. CLSI EP17-A**24**, 34 (2004).

[44] Gardner, R. C. et al. Cavum septum pellucidum in retired American Pro-Football players. *J. Neurotrauma*. **33** (1), 157–161 (2016). 25970145 10.1089/neu.2014.3805 PMC4696427

[45] Desikan, R. S. et al. An automated labeling system for subdividing the human cerebral cortex on MRI scans into gyral based regions of interest. *Neuroimage* **31** (3), 968–980 (2006). 10.1016/j.neuroimage.2006.01.021 16530430

[46] Dickerson, B. C. et al. The cortical signature of Alzheimer’s disease: regionally specific cortical thinning relates to symptom severity in very mild to mild AD dementia and is detectable in asymptomatic amyloid-positive individuals. *Cereb. Cortex*. **19** (3), 497–510 (2009). 18632739 10.1093/cercor/bhn113 PMC2638813

[47] van Engelen, M. E. et al. Altered brain metabolism in frontotemporal dementia and psychiatric disorders: involvement of the anterior cingulate cortex. *EJNMMI Res.* **13** (1), 71 (2023). 37493827 10.1186/s13550-023-01020-2 PMC10371967

[48] Lu, P. H. et al. Patterns of brain atrophy in clinical variants of frontotemporal Lobar degeneration. *Dement. Geriatr. Cogn. Disord*. **35** (1–2), 34–50 (2013). 23306166 10.1159/000345523 PMC3609420

[49] Montenigro, P. H. et al. Clinical subtypes of chronic traumatic encephalopathy: literature review and proposed research diagnostic criteria for traumatic encephalopathy syndrome. *Alzheimers Res. Ther.* **6** (5), 68 (2014). 25580160 10.1186/s13195-014-0068-z PMC4288217

[50] Macbeth, G., Razumiejczyk, E. & Ledesma, R. Cliff’s Delta calculator: A non-parametric effect size program for two groups of observations. *Universitas Physiol.* **10**, 545–555 (2011).

[51] Verhage, F. *Intelligentie En Leeftijd: Onderzoek Bij Nederlanders Van Twaalf Tot Zevenenzeventig Jaar. Bijdragen Tot De Psychologie* (Van Gorcum, 1964).

