# Supplementary material for: Plasma and MRI biomarkers capture neuronal damage in former professional boxers
Source: Sci Rep. 2025 Jun 6;15:20005. doi: 10.1038/s41598-025-93066-6 (PMC12144257; doi:10.1038/s41598-025-93066-6)
Supplement: Supplementary file 1 — Supplementary Material 1 [file 41598_2025_93066_MOESM1_ESM.docx]

**Plasma and MRI Biomarkers capture Neuronal Damage in Former Professional Boxers - Data supplement**

S. Kamps, S. van Amerongen, E. R. Blujdea, M. Hoppen, B. Bongers, K.H. Schelvis, D.K. Caton, M. Königs, C.E. Teunissen, P. Scheltens, R. Ossenkoppele, E.G.B. Vijverberg

*
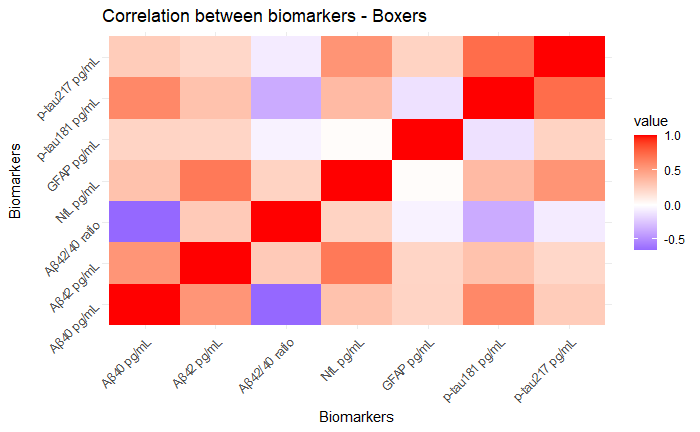
*

**A.**

*
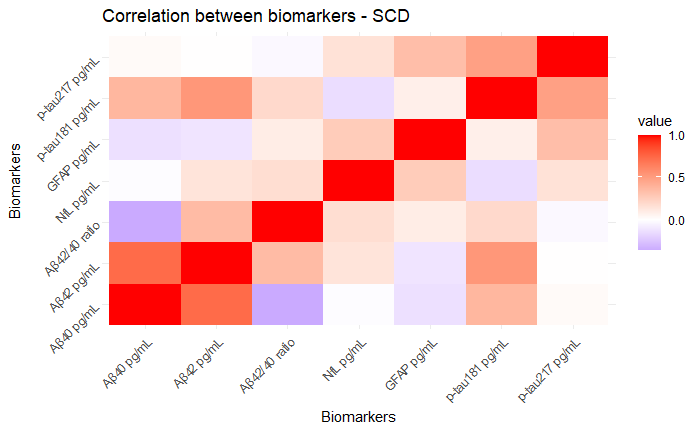
*

**B.**

*
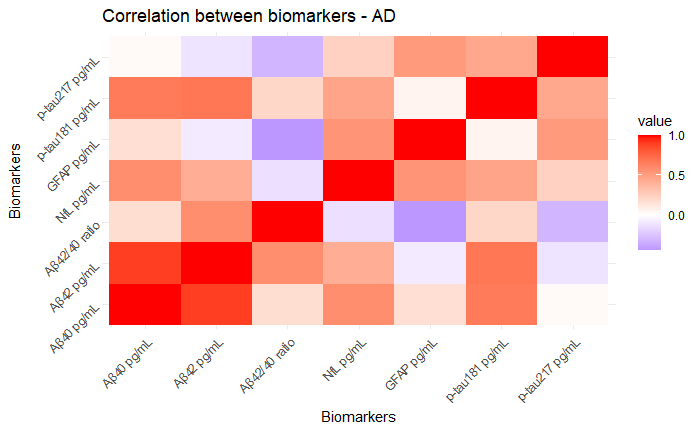
*

**C.**

**Figure S1.** Heatmaps showing correlations between each plasma biomarker (in pg/mL unit) for each of the groups. A) Boxers, B) SCD, C) AD. Abbreviations: Aβ = amyloid beta , GFAP = Glial Fibrillary Acidic Protein , NfL = neurofilament light , p-tau = phosphorylated tau, AD=Alzheimer’s Disease, SCD=Subjective Cognitive Decline


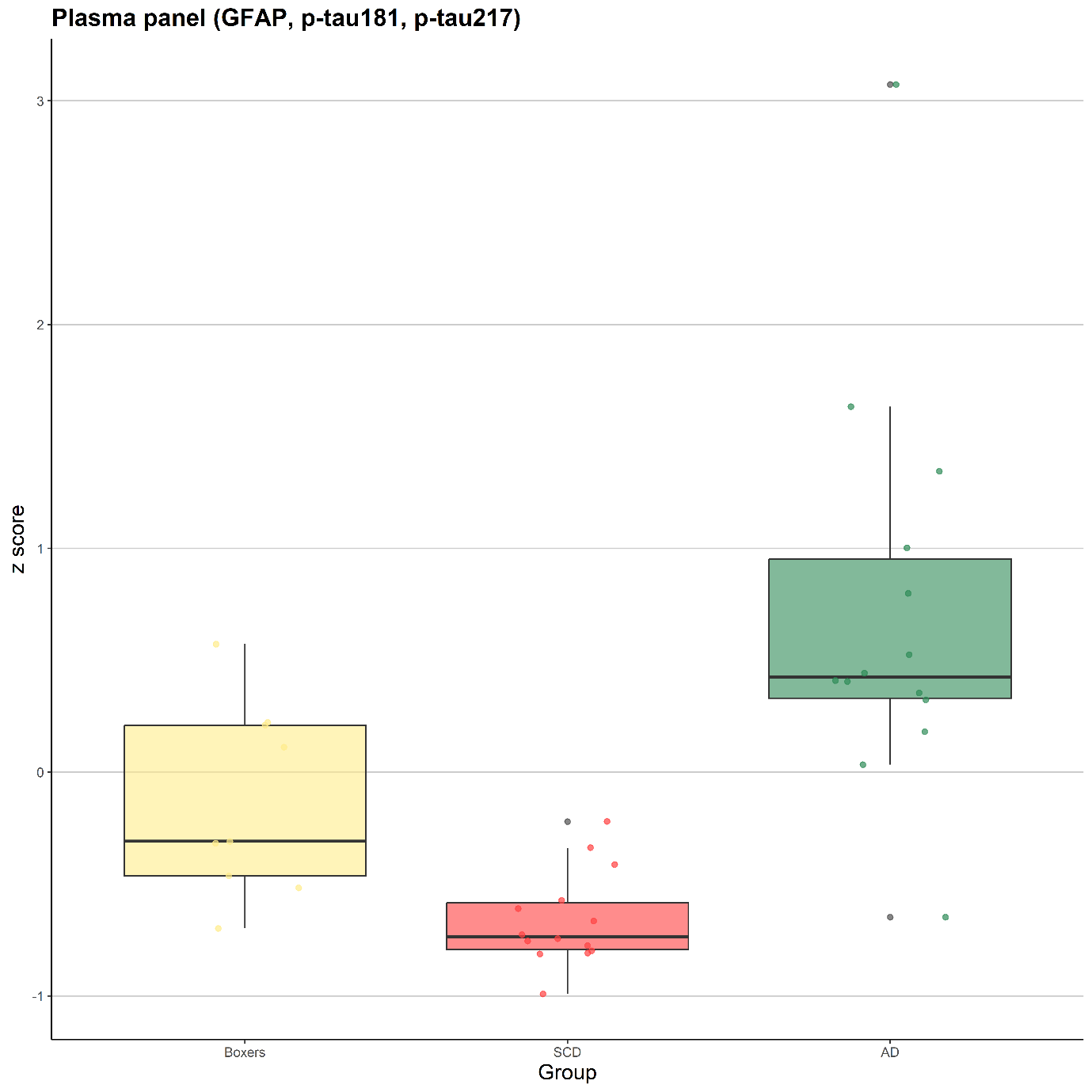


**Figure S2.** Boxplot for the combined plasma panel (GFAP, p-tau181, p-tau217) between groups. Z-scores were calculated within the study sample. Kruskal-Wallis tests showed group differences on the plasma panel (χ²=23.45, p<.001). Boxers had higher plasma panel scores compared to SCD (δ=.778 [CI= .092, .963], p=.002) and lower concentrations compared to AD (δ=-.667 [CI=-.892, .177], p=.007). Abbreviations: AD=Alzheimer’s Disease, SCD=Subjective Cognitive Decline

**Figure S2.**

**
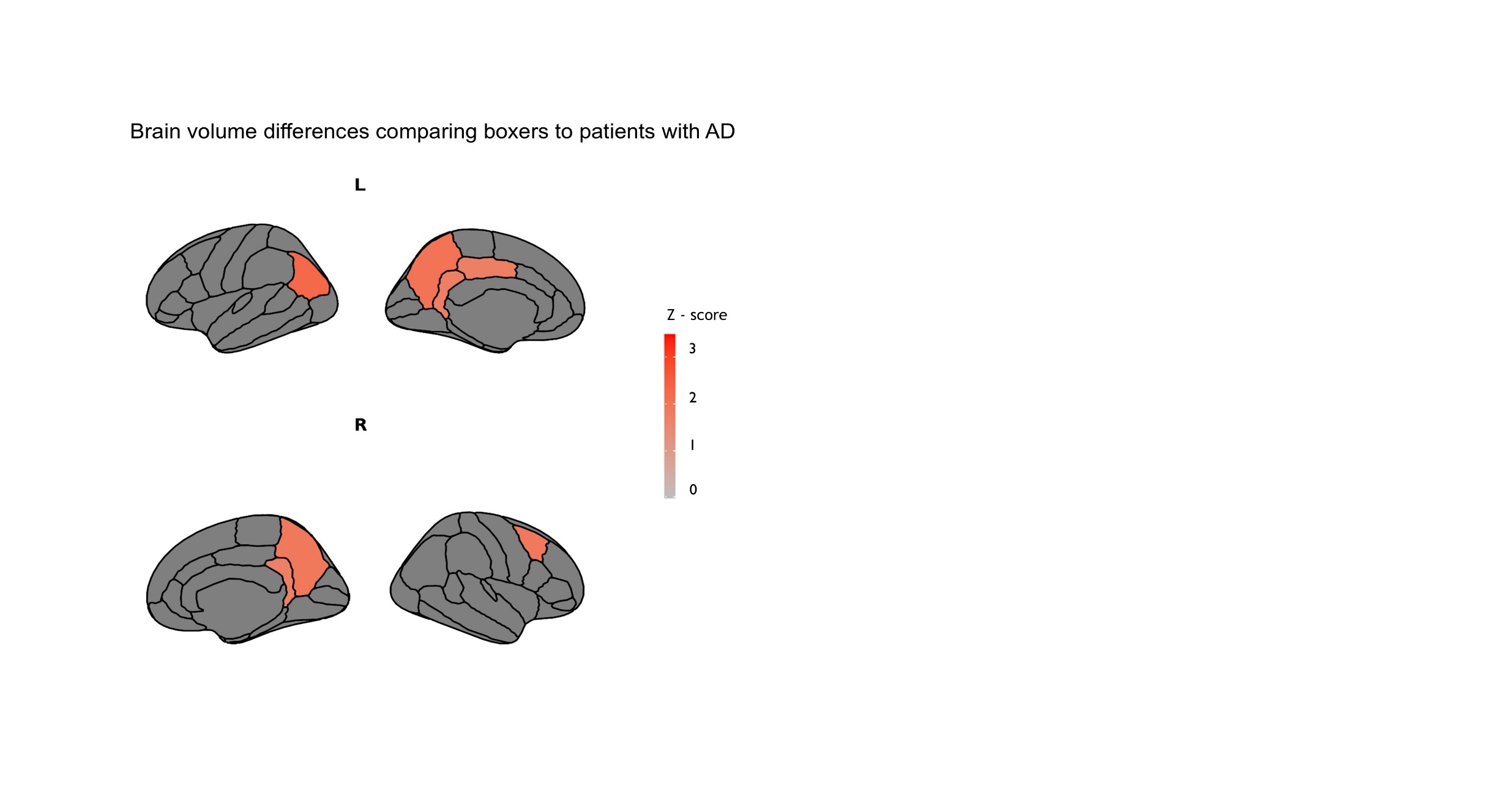
**

**Figure S3. Corticalbrain volume differences within the pre-specified disease-specific ROIs comparing boxers to patients with AD.** Brain volumes of pre-specified disease-specific regions of interest (ROI’s) were derived from Freesurfer and values were z-transformed. Regions were based on the Desikan-Killiany (DK) atlas. Differences in the z-transformed volumetric values for the regions between groups were tested with t-tests. All p-values are adjusted to correct for multiple testing and the False Discovery Rate (FDR). Z-scores are indicated by the heat map on the right. The legend contains only positive Z-scores, this is due to the finding that boxers had only significantly higher volumes than patients with AD, and not lower. The brain maps are plotted with the ggseg package in R. Regions that differed between boxers and patients with SCD, i.e. regions that showed significantly different volumes in boxers compared to patients with AD, are colored in the brain maps. Darker colors indicate stronger differences between groups. Non-significant values are not displayed. Significant differences between groups were found for the following regions: precuneus (bilateral), isthmus cingulate (bilateral), posterior cingulate (left), inferior parietal (left), caudal middle frontal (right). Between boxers and patients with AD, no significant differences in subcortical brain volumes within the disease-specific ROI’s were found, therefore only the cortical (dk) map is displayed. Abbreviations: AD=Alzheimer’s Disease, L=left, R=right

**
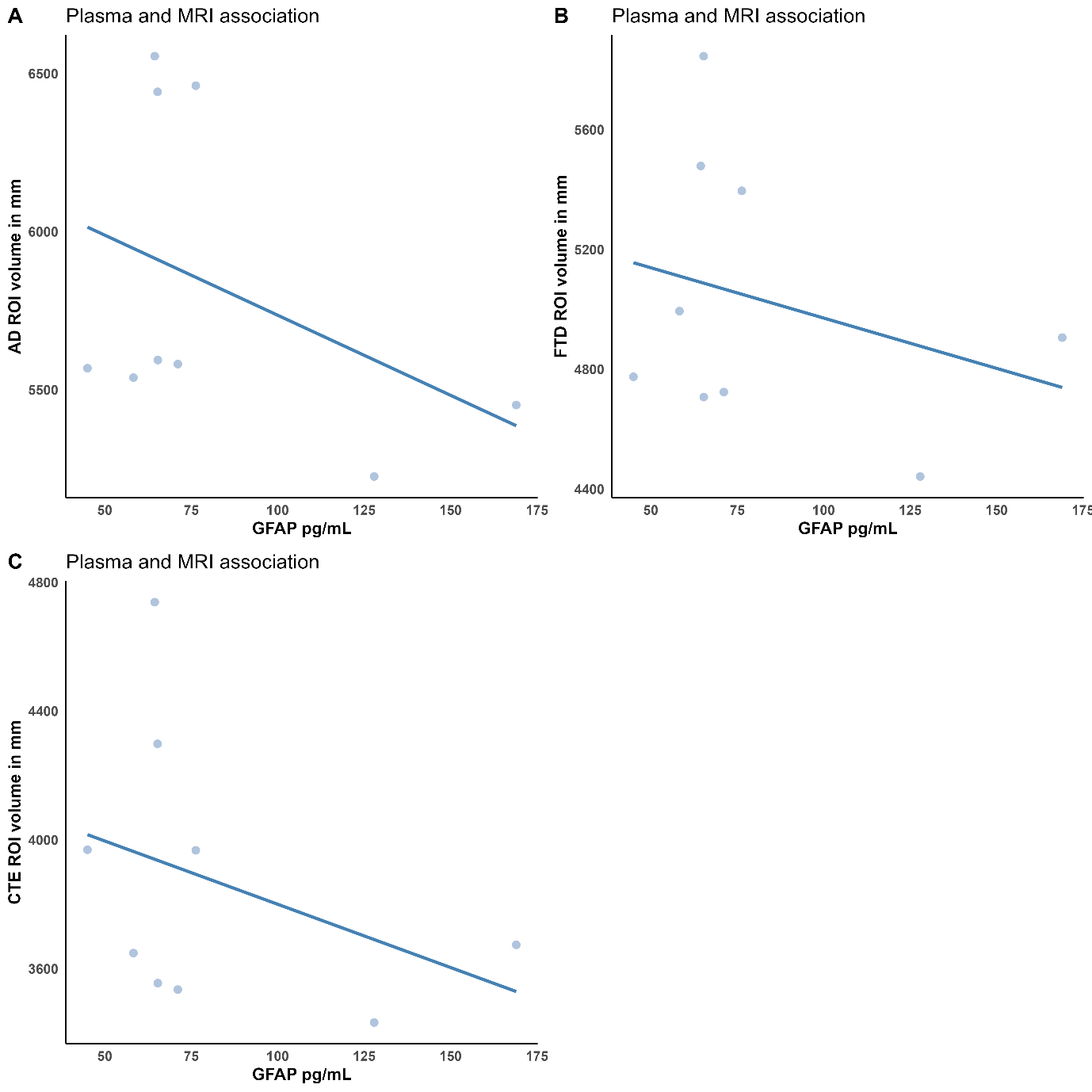
**

**Figure S4.** Regression plots for the non-significant associations between plasma GFAP and disease-specific ROI volumes. A) AD-related= entorhinal, inferior temporal, temporal pole, inferior parietal, superior frontal, superior parietal, supramarginal, precuneus, inferior frontal. B) FTD-related= insula, rostral anterior frontal, caudal anterior frontal, superior frontal, superior parietal, medial orbitofrontal, lateral orbitofrontal. C) CTE-related= hippocampus, amygdala, thalamus, entorhinal, inferior parietal, superior temporal, medial orbitofrontal, lateral orbitofrontal. Abbreviations**:** AD = Alzheimer’s disease, CTE = chronic traumatic encephalopathy, FTD = frontotemporal dementia, ROI = Region of Interest

| **Table S1.** Descriptive statistics | | | | |
| --- | --- | --- | --- | --- |
|  | AD | SCD | Boxers | p |
| n | 15 | 14 | 9 |  |
| Smoking yes/no (%) | |  |  | 0.024 |
| no | 5 (38.5) | 7 (50.0) | 7 (77.8) |  |
| used to | 7 (53.8) | 2 (14.3) | 0 (0.0) |  |
| yes | 1 (7.7) | 5 (35.7) | 2 (22.2) |  |
| hypercholesterolomy = yes (%) | 13 (92.9) | 10 (83.3) | 7 (100.0) | 0.450 |
| hypertension = no (%) | 9 (64.3) | 9 (75.0) | 7 (100.0) | 0.197 |
| familiar cardiovascular disease = no (%) | 4 (33.3) | 5 (38.5) | 6 (66.7) | 0.274 |
| familiar dementia = no (%) | 8 (66.7) | 6 (46.2) | 6 (66.7) | 0.498 |
| familar psychiatry = no (%) | 10 (83.3) | 9 (69.2) | 7 (77.8) | 0.704 |
| Digit span forward score (mean (SD)) | 10.79 (3.72) | 13.36 (2.56) | 12.11 (2.71) | 0.104 |
| Digit span backward score (mean (SD)) | 6.43 (2.90) | 8.57 (1.79) | 7.89 (2.37) | 0.070 |
| VAT naming (mean (SD)) | 11.73 (0.59) | 12.00 (0.00) | 11.00 (1.94) | 0.074 |
| RAVLT trial 1 (mean (SD)) | 2.67 (1.29) | 5.29 (1.82) | 3.67 (1.22) | <0.001 |
| RAVLT trial 2 (mean (SD)) | 3.87 (1.46) | 8.29 (2.67) | 5.22 (0.97) | <0.001 |
| RAVLT trial 3 (mean (SD)) | 4.07 (1.49) | 9.43 (3.20) | 6.33 (1.32) | <0.001 |
| RAVLT trial 4 (mean (SD)) | 4.43 (1.09) | 10.14 (3.32) | 8.00 (1.22) | <0.001 |
| RAVLT trial 5 (mean (SD)) | 5.07 (2.20) | 10.36 (3.23) | 8.00 (1.80) | <0.001 |
| RAVLT delayed recall (mean (SD)) | 1.21 (1.72) | 8.29 (3.95) | 5.11 (2.15) | <0.001 |
| RAVLT false negatives (mean (SD)) | 2.71 (2.46) | 1.07 (1.54) | 5.67 (5.89) | 0.012 |
| RAVLT trial false positives (mean (SD)) | 5.14 (3.61) | 0.36 (0.93) | 6.11 (6.49) | 0.002 |
| VAT A trial 1 (mean (SD)) | 2.36 (2.56) | 5.21 (1.25) | 5.22 (0.97) | <0.001 |
| VAT A trial 2 (mean (SD)) | 3.00 (2.75) | 5.71 (0.83) | 6.00 (0.00) | <0.001 |
| VAT A trial 3 (mean (SD)) | 6.00 (0.00) | 5.79 (0.58) | 6.00 (0.00) | 0.384 |
| Letterfluency letter D (mean (SD)) | 9.92 (4.11) | 12.14 (5.19) | 10.00 (6.16) | 0.463 |
| Letterfluency letter A (mean (SD)) | 8.23 (3.98) | 10.21 (3.38) | 10.75 (5.70) | 0.333 |
| Letterfluency letter T (mean (SD)) | 10.46 (3.55) | 11.43 (3.65) | 12.75 (4.23) | 0.407 |
| Abbreviations: VAT=Visual Association Test, RAVLT=Raven Auditory Verbal Learning Test, AD=Alzheimer’s Disease, SCD=Subjective Cognitive Decline | | | | |

| **Table S2**. Longitudinal data for the five boxers | | | | | | | | | | | | |  |  |
| --- | --- | --- | --- | --- | --- | --- | --- | --- | --- | --- | --- | --- | --- | --- |
| Boxer | Age range | Follow-up duration | Time | MMSE | Aβ42 pg/mL | Aβ42/40 ratio | GFAP pg/mL | NfL pg/mL | P-tau181 pg/mL | P-tau217 pg/mL | Memory composite z score | EF composite z score | |  |
| 1 | 45-55 | 7 years | BL | 27 | 7.16 | 0.074 | 76.31 | 8.30 | 2.23 | 0.35 | 0.65 | 1.11 | |  |
|  |  |  | FU | 27 | 7.30 | 0.071 | 75.9 | 11.0 | 2.86 | 0.41 | -0.51 | 0.53 | |  |
| 2 | 52-62 | 7 years | BL | 27 | 5.99 | 0.067 | 65.26 | 7.92 | 1.50 | 0.32 | -1.32 | -0.05 | |  |
|  |  |  | FU | 29 | 7.14 | 0.064 | 66.6 | 10.8 | 2.36 | 0.34 | -2.13 | -0.73 | |  |
| 3 | 52-62 | 7 years | BL | 26 | 6.75 | 0.078 | 44.96 | 11.63 | 2.21 | 0.40 | -0.36 | 0.01 | |  |
|  |  |  | FU | 26 | 7.88 | 0.072 | 59.7 | 16.2 | 2.55 | 0.56 | -1.13 | 0.21 | |  |
| 4 | 50-60 | 7 years | BL | 26 | 5.74 | 0.064 | 58.27 | 12.31 | 2.01 | 0.58 | -1.01 | 0.68 | |  |
|  |  |  | FU | 29 | 5.75 | 0.064 | 127.0 | 17.3 | 3.26 | 0.91 | -3.18 | -0.28 | |  |
| 5 | 70-80 | 6 years | BL | 27 | 6.42 | 0.062 | 65.32 | 8.69 | 1.02 | 0.15 | -0.83 | -0.16 | |  |
|  |  |  | FU | 26 | 8.68 | 0.070 | 81.8 | 11.0 | 1.08 | 0.20 | -0.52 | -1.45 | |  |
| Mean (SD) or median [IQR]* | 57.8 | NA | NA | 24.79 (4.57) | 6.03 (1.29) | 0.06 (0.01) | 85.26 (59.29) | 13.54 (6.94) | 2.17 [1.51, 3.23] | 0.49 [0.26, 0.85) | -1.90 (2.49) | -0.54 (0.82) | |  |
| The mean changes per year from ‘baseline’ (time of first measurement) to follow-up were as follows: Aβ40: 14.51 pg/mL; Aβ42: 0.94 pg/mL; Aβ42/40: -0.0009; GFAP: 20.18 pg/mL; NfL; 3.49 pg/mL; p-tau181: 0.63 pg/mL; p-tau217: 0.12 pg/mL. For privacy protective reasons, ages are shown in ranges, with the follow-up time in years provided. Abbreviations: BL = baseline, FU = follow-up, MMSE = Mini Mental State Examination , EF = executive functioning , Aβ = amyloid beta , GFAP = Glial Fibrillary Acidic Protein , NfL = neurofilament light , p-tau = phosphorylated tau. * Scaled across whole study sample | | | | | | | | | | | | | | |
